# Pharmacokinetics, biodistribution and brain retention of a bispecific antibody-based PET radioligand for imaging of amyloid-β

**DOI:** 10.1038/s41598-017-17358-2

**Published:** 2017-12-08

**Authors:** Dag Sehlin, Xiaotian T. Fang, Silvio R. Meier, Malin Jansson, Stina Syvänen

**Affiliations:** Department of Public Health and Caring Sciences/Geriatrics, Uppsala University, Rudbeck Laboratory, Dag Hammarskjölds väg 20, SE-751 83 Uppsala, Sweden

## Abstract

Monoclonal antibodies (mAbs) have not been used as positron emission tomography (PET) ligands for *in vivo* imaging of the brain because of their limited passage across the blood-brain barrier (BBB). However, due to their high affinity and specificity, mAbs may be an attractive option for brain PET if their brain distribution can be facilitated. In the present study, a F(ab’)_2_ fragment of the amyloid-beta (Aβ) protofibril selective mAb158 was chemically conjugated to the transferrin receptor (TfR) antibody 8D3 to enable TfR mediated transcytosis across the BBB. The generated bispecific protein, 8D3-F(ab’)_2_-h158, was subsequently radiolabeled and used for microPET imaging of Aβ pathology in two mouse models of AD. [^124^I]8D3-F(ab’)_2_-h158 was distributed across the BBB several fold more than unmodified mAbs in general and its accumulation in the brain reflected disease progression, while its concentration in blood and other organs remained stable across all age groups studied. Cerebellum was largely devoid of 8D3-F(ab’)_2_-h158 in young and middle aged mice, while mice older than 18 months also showed some accumulation in cerebellum. In a longer perspective, the use of bispecific antibodies as PET ligands may enable *in vivo* ‘immunohistochemistry’ also of other proteins in the brain for which PET radioligands are lacking.

## Introduction

**Figure 1:**
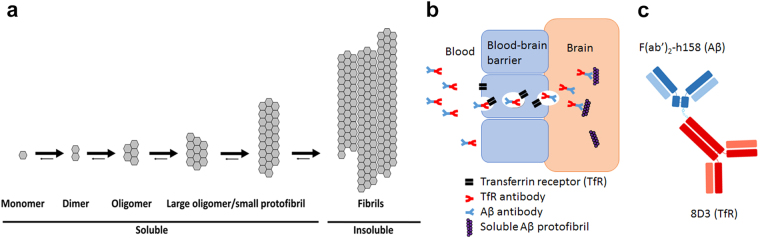
Aggregation of the Aβ peptide and receptor mediated transcytosis across the BBB. (**a**) If insufficiently cleared from the brain, monomeric Aβ becomes misfolded and prone to aggregate. (**b**) The transferrin receptor (TfR) can be used as a shuttle for antibodies across the BBB if the antibody incorporates a moiety that binds to the TfR in a reversible mode. Here, this strategy was applied using a bispecific antibody based on the TfR binding antibody 8D3 conjugated to a F(ab’)_2_ fragment of the Aβ protofibril selective antibody mAb158 (**c**).

Alzheimer’s disease (AD) is characterized by deposition of amyloid-beta (Aβ) in the brain. The Aβ peptide, when present in excess in the brain, becomes misfolded and starts to aggregate into larger assemblies that gradually become less soluble (Fig. 1a). The insoluble Aβ plaques can be visualized with the positron emission tomography (PET) radioligand [^11^C]PIB^1^. PET imaging with [^11^C]PIB has been an important improvement for diagnosing AD, especially in ruling out AD and in cases where the cause of dementia is unclear^2^. However, the total plaque load, quantified with [^11^C]PIB and other small molecular radioligand analogues, does not reflect disease stage well as it appears that the PET signal becomes saturated rather early during the disease progression^3,4^. Further, therapeutic interventions in clinical trials are currently explored in patients at early disease stages and are, among other targets, directed at earlier, still soluble, forms of aggregated Aβ, e.g. oligomers and protofibrils, as these have been indicated as the neurotoxic form of Aβ^5–10^. In addition, although the exact fraction is unknown, some AD patients lack dense core plaques and may therefore be falsely diagnosed as Aβ negative with [^11^C]PIB and analogues^11^. Thus, this highlights the need for a PET radioligand that can visualize other than insoluble forms of Aβ aggregates.

Monocolonal antibodies (mAbs) are in general highly specific for their target which is an essential feature of a PET radioligand as the specific-to-nonspecific signal is the crucial parameter to optimize for good quality PET images and quantification. However, their use as PET radioligands for intra-brain targets is limited due to their low and slow passage across the blood-brain barrier (BBB). We and others have previously shown that transport of mAbs across the BBB can be enhanced by introducing specificity also for the transferrin receptor (TfR)^12–15^ with protein engineering. The TfR is expressed at the brain capillary endothelial cells and can be used as a shuttle between the luminal and abluminal side of the BBB (Fig. 1b). We have previously described the use of this mechanism to increase the brain concentrations of the monoclonal antibody mAb158, which binds selectively to soluble Aβ protofibrils, with only moderate binding to fibrillar Aβ, low binding to Aβ monomers and no binding the Aβ protein precursor (AβPP)^16,17^. This specificity is due to a relatively low affinity interaction with N-terminal amino acids of the Aβ monomer, but a high avidity, bivalent binding to the polymeric structure of the Aβ protofibril. When further aggregated, epitopes are partially hidden, which reduces the binding strength to fibrillar Aβ. While soluble Aβ protofibrils are less abundant than the dense amyloid plaques, in the AD brain as well as in transgenic mice, they are probably more accessible for interactions with mAbs penetrating the brain. Thus, with radiolabeled variants of modified mAb158 we could visualize Aβ protofibrils *in vivo* with preclinical microPET imaging, displaying a close correlation with biochemically measured brain levels of Aβ protofibrils^15,17,18^.

In the present study the aim was to investigate the brain accumulation of the novel bispecific mAb-based radioligand [^124^I]8D3-F(ab’)_2_-h158 (Fig. 1c) in wild-type (wt) and two transgenic models of Aβ pathology displaying different disease progression patterns: tg-ArcSwe mice, harboring both the Arctic and the Swedish AβPP mutations, display an early onset of Aβ pathology with dense Aβ deposits, resembling human amyloid plaques; tg-Swe mice overexpress AβPP with only the Swedish mutation and has a late onset of Aβ pathology but with a rapid progression and less dense deposits^19,20^. Mice aged 4 to 24 months, i.e. a large age span, were included in order to cover the initial stages of Aβ accumulation, the deposition of the first dense core plaques and the late disease progression stages when the brain contains very large amounts of all forms of aggregated Aβ. The two mouse models, with different manifestations of Aβ pathology, were chosen to obtain a broader understanding of the properties of the PET ligand. In addition, systemic pharmacokinetics and distribution to major peripheral organs were also investigated.

## Results

### Radiolabeling and injections

The purity and stability of radioiodinated 8D3-F(ab’)_2_-h158 was assessed with instant thin layer chromatography (ITLC). Radioligand purity was 96% directly after labeling and after a 72 h incubation in mouse blood plasma, 86% of the radioactivity was found in the high molecular weight fraction, indicating a relatively low degree of deiodination under *in vivo*-like conditions. For PET experiments, [^124^I]8D3-F(ab’)_2_-h158 was obtained with a labeling yield of 72.4 ± 5.5% and a specific activity of 218 ± 22 MBq/nmole. Mice were injected with 12.3 ± 3.2 MBq [^124^I]8D3-F(ab’)_2_-h158 three days before PET imaging.

### Pharmacokinetics and biodistribution

**Figure 2:**
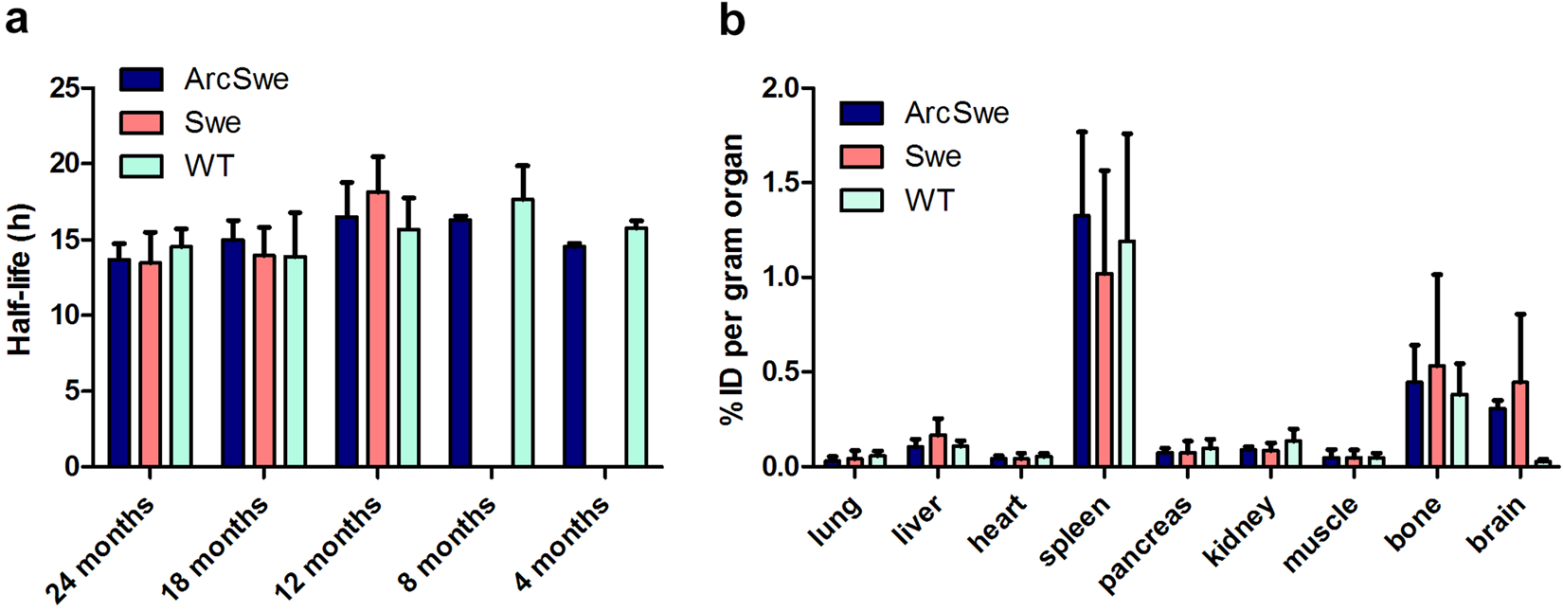
Half-life in blood and biodistribution of [^124^I]8D3-F(ab’)_2_-h158. (**a**) Elimination from blood was independent of genotype and age (n ≥ 3 in all groups except tg-ArcSwe 4 and 8 months, tg-Swe 12 months and wt 4 months, where n = 2). (**b**) Concentration of [^124^I]8D3-F(ab’)_2_-h158 in peripheral organs 72 h after administration in transgenic (tg-ArcSwe, 8–24 months, n = 9; tg-Swe, 12–24 months, n = 8) and wt mice (8–24 months, n = 7). Concentration in brain tissue in old mice (tg-ArcSwe, 18–24 months, n = 7; tg-Swe, 18–24 months, n = 5; wt, 18–24 months, n = 7) are included to allow for comparison. In all cases data is represented as mean ± standard deviation.

After administration, [^124^I]8D3-F(ab’)_2_-h158 showed a fairly rapid distribution phase; at 24 h radioligand concentration in blood was about 25% of that observed at 3 h. The half-life in blood calculated from 3 h to 72 h post injection was 15.1 ± 2.1 h. There was no difference in radioligand half-life between transgenic and wt mice at any age. In addition, there was no influence of age on blood pharmacokinetics (Fig. 2a).

Genotype and age did not influence the distribution to peripheral organs. In general, concentrations in major organs were low with the exception of spleen and bone (probably bone marrow) that showed concentrations of 1.1 ± 0.5%ID/g and 0.5 ± 0.3% ID/g, respectively (Fig. 2b). The brain concentrations were 0.31 ± 0.05, 0.44 ± 0.4 and 0.03 ± 0.01%ID/g at 72 h after administration in 18–24 months old tg-ArcSwe, tg-Swe and wt animals, respectively. Thus, the transgenic animals displayed 10–15-fold higher brain concentrations of [^124^I]8D3-F(ab’)_2_-h158 than wt animals at this time point and 5–10 fold higher concentrations than anticipated for unmodified IgG antibodies^21^.

### PET

**Figure 3:**
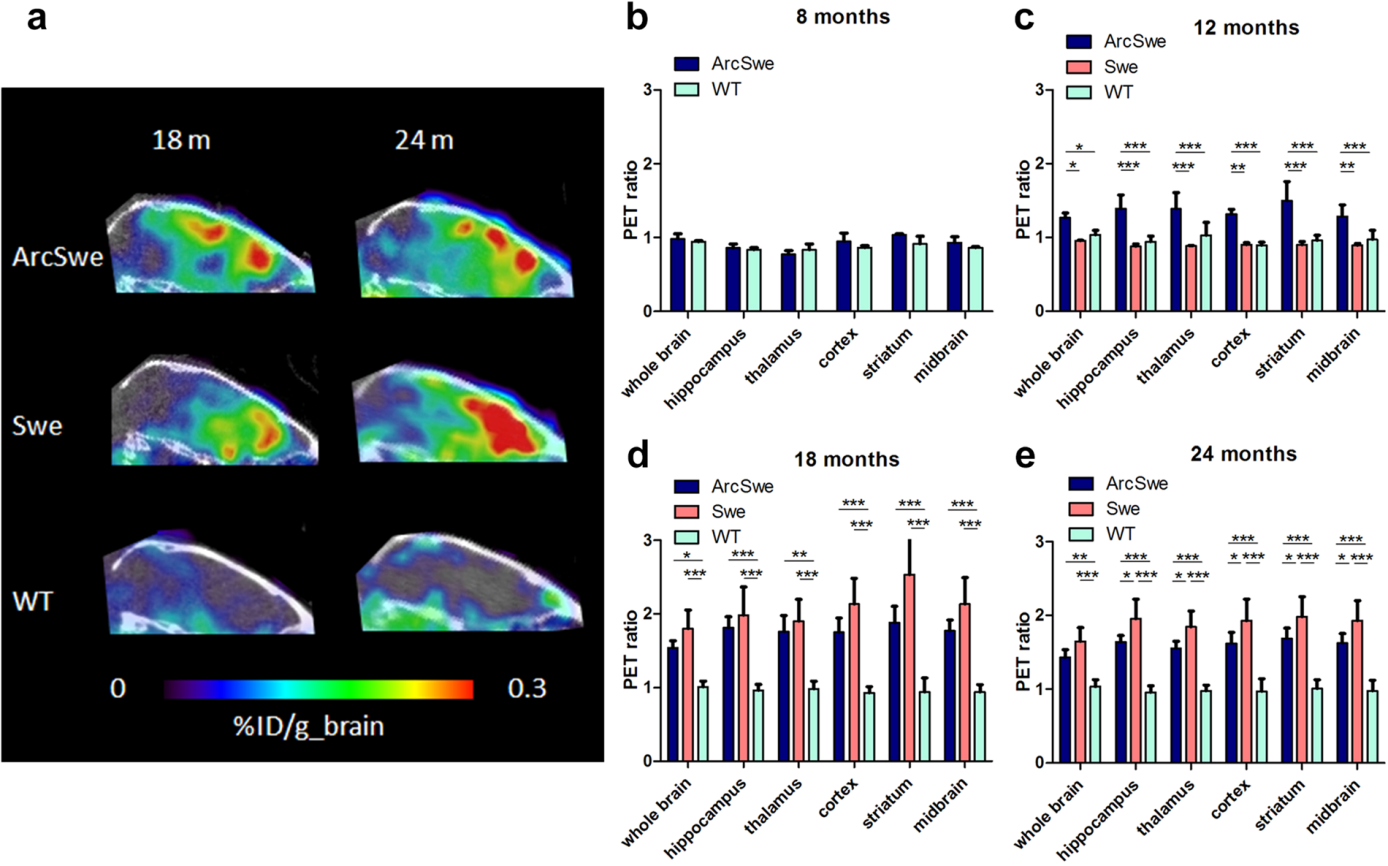
PET imaging in tg-ArcSwe, tg-Swe and wt mice at different ages. (**a**) Sagittal PET images of 18 and 24 months old mice. Quantifiaction of PET imaging, displaying a PET concentration ratio (region of interest/cerebellum) in 8 months old tg-ArcSwe (n = 2) and wt (n = 3) mice (**b**); 12 months old tg-ArcSwe (n = 4), tg-Swe (n = 2) and wt (n = 5) mice (**c**); 18 months old tg-ArcSwe (n = 5), tg-Swe (n = 13) and wt (n = 4) mice (**d**); and in 24 months old tg-ArcSwe (n = 5), tg-Swe (n = 3) and wt (n = 4) mice (**e**). Representative images are shown in (**a**); data is represented as mean ± standard deviation in (**b**–**e**). Significant differences between groups were tested with two-way ANOVA followed by Bonferroni’s *post hoc* test (*p < 0.05, **p < 0.1, ***0.001 p < 0.001).

PET imaging revealed clear differences between transgenic mice and wt mice. Regardless of age, wt mice displayed a very low signal in the brain area. Tg-Swe mice, with a late onset and a fairly rapid disease progression, showed Aβ pathology that could be visualized with PET at the age of 18 months, while tg-ArcSwe mice, with an earlier onset of pathology, displayed a signal considerably higher than that observed in wt mice already at the age of 12 months. The PET signal in tg-Swe, but not in tg-ArcSwe, increased between the age of 18 and 24 months (Fig. 3a). Quantification using cerebellum as a reference region showed no significant differences in the PET concentration ratio (region of interest/cerebellum) between tg-ArcSwe and wt animals the in 8 months old group (Fig. 3a). Tg-ArcSwe animals displayed a significantly higher ratio than tg-Swe and wt animals in all regions in the 12 month group, but there was no difference between tg-Swe and wt animals (Fig. 3c). At 18 months of age, both tg-ArcSwe and tg-Swe mice showed significantly higher PET ratios in all regions compared to wt mice and there were no differences between tg-ArcSwe and tg-Swe except in the striatum where tg-Swe displayed a significantly higher ratio (Fig. 3d). Although the PET images indicated a more intense signal at 24 months compared to 18 months in tg-Swe, the PET concentration ratio was not increased in 24 month old animals compared to 18 month old animals in any of the studied regions. However, at 24 months, tg-Swe mice showed higher ratios than tg-ArcSwe in all regions except whole brain (Fig. 3e).

### Correlations between age, *in vivo* and *ex vivo* measured [^124^I]8D3- F(ab’)_2_-158 concentrations

**Figure 4:**
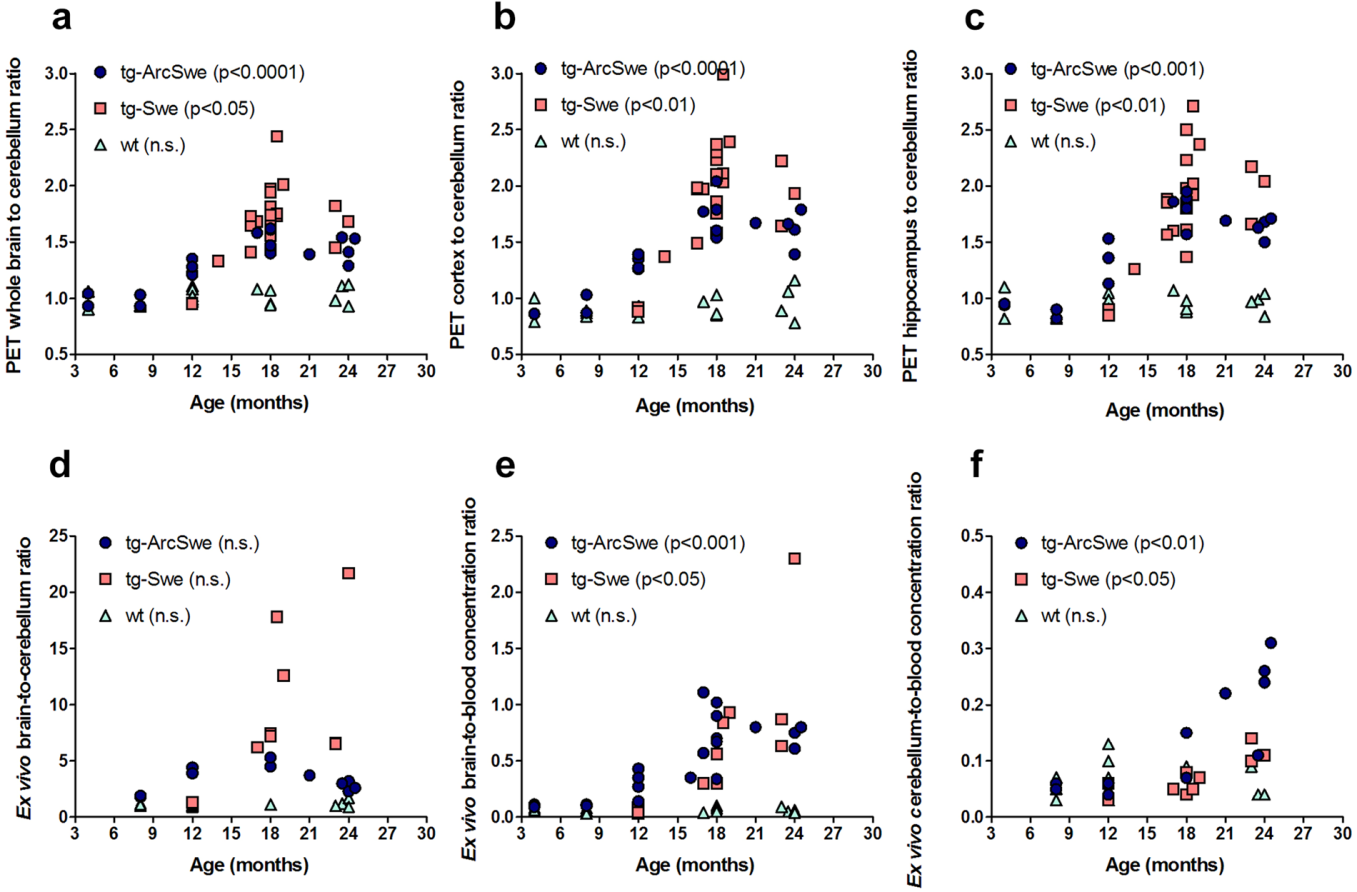
Brain concentrations and pathology progression. Correlation analysis of age and brain concentration of [^124^I]8D3-F(ab’)_2_-h158 expressed as a region of interest-to cerebellum ratio, determined with PET (**a**–**c**, tg-ArcSwe, n = 18; tg-Swe, n = 22; wt, n = 18) and *ex vivo* radioactivity measurements (**d**, tg-ArcSwe, n = 11; tg-Swe, n = 10; wt, n = 14). *Ex vivo* brain-to-blood and cerebellum-to-blood ratios are given in (**e**; tg-ArcSwe, n = 20; tg-Swe, n = 10; wt, n = 18) and (**f**; tg-ArcSwe, n = 11; tg-Swe, n = 10; wt, n = 13) respectively. *N*. *B*. the different y-axis scale in (**e**) and (**f**) due to much lower concentration of [^124^I]8D3-F(ab’)_2_-h158 in cerebellum compared to the rest of the brain. Pearson’s correlation was used to analyze correlation of brain concentration of [^124^I]8D3-F(ab’)_2_-h158 and age or Aβ protofibril concentration (**a**–**f**).

In line with the development of Aβ pathology during disease progression, the concentration of [^124^I]8D3-F(ab’)_2_-h158 increased with age in the transgenes. Wild type mice, without Aβ pathology, showed very low concentrations of [^124^I]8D3-F(ab’)_2_-h158 in all studied brain regions regardless of age. In earlier stages of Aβ pathology development, the concentrations increased in affected brain regions of both tg-ArcSwe and tg-Swe, but not in cerebellum which is a region usually considered to be largely unaffected by Aβ pathology. Thus, there was a significant correlation between the PET brain-to-cerebellum ratio and age for both tg-ArcSwe (p < 0.0001) and tg-Swe (p = 0.0116) mice, while no such correlation was found for wt mice (p = 0.23) (Fig. 4a). The same was true for the cortex-to-cerebellum and hippocampus-to-cerebellum ratios (Fig. 4b,c). However, in very old mice, aged around 24 months, these ratios tended to display a plateau or even decline, most evident in tg-ArcSwe mice. *Ex vivo* quantification of the brain-to-cerebellum ratio (Fig. 4d) showed a similar pattern, with an even more pronounced decline in old tg-ArcSwe mice compared to the *in vivo* quantification with PET, and Pearson’s correlation for this group was therefore not significant (p = 0.77). The brain-to-blood concentration ratio was also significantly correlated with age in transgenes (p < 0.001 tg-ArcSwe, p = 0.012 tg-Swe) while this was not the case in wt mice (p = 0.65) (Fig. 4e). Although lower than in the rest of the brain, increased levels of [^124^I]8D3-F(ab’)_2_-h158 were observed also in cerebellum, in tg-ArcSwe from the age of 18 months and in tg-Swe in the very oldest group of mice, i.e. at 24 months of age (Fig. 4f).

### Histopathological analyses

**Figure 5:**
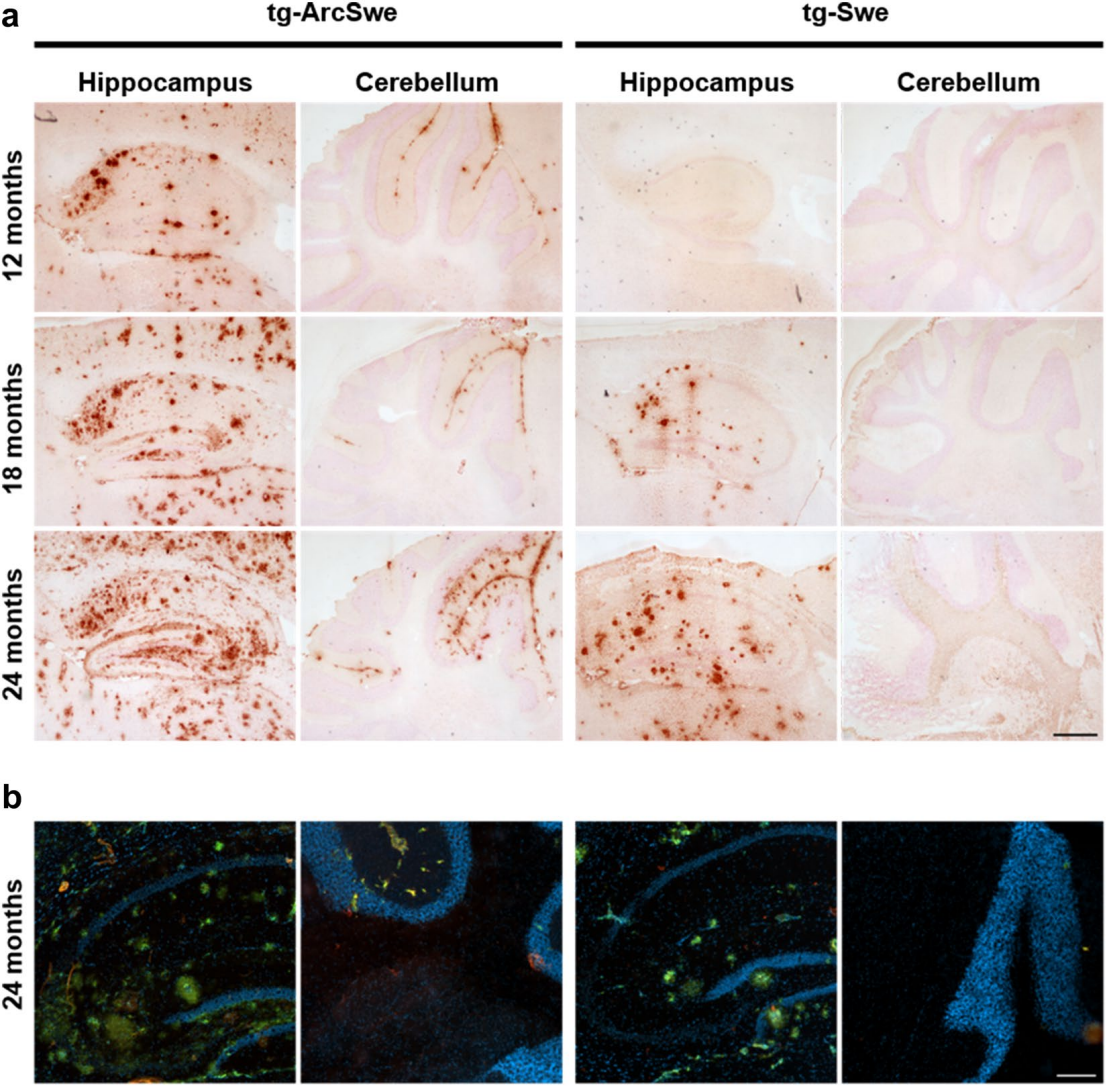
Histopathological analyses. Aβ pathology visualized in the hippocampus and cerebellum of tg-ArcSwe (n = 6; 2 images/region) and tg-Swe (n = 8; 2 images/region) mice at different ages. (**a**) Aβ40 staining, in the hippocampus and cerebellum of 12–24 month old tg-ArcSwe and tg-Swe mice. Scale bar, 1000 µm. (**b**) Aβ pathology visualized with Amytracker^TM^ in 24 months old tg-ArcSwe and tg-Swe mice. Scale bar, 200 µm.

Aβ pathology was visualized in brain sections from a subset of the tg-ArcSwe and tg-Swe mice with both Aβ40 immunohistochemistry and Amytracker^TM^ staining. Both histological methods confirmed increased Aβ pathology in cortex and hippocampus with age, including an earlier onset of pathology in tg-ArcSwe compared to tg-Swe mice (Fig. 5). The cerebellum was largely devoid of Aβ staining in tg-Swe mice of all ages, while tg-ArcSwe mice started to display pathology that could be stained with Aβ40 immunohistochemistry already at an age of 12 months (Fig. 5a) and with Amytracker^TM^ at an age of 18 months (Fig. 5b).

## Discussion

In the present study we have investigated the spatial brain distribution of the novel antibody-based PET radioligand [^124^I]8D3-F(ab’)_2_-h158, its biodistribution to peripheral organs, blood pharmacokinetics and how these characteristics correlate with age and Aβ pathology in two different mouse models of AD and age matched wt mice without Aβ pathology. Mice of a wide range of ages were used to enable analysis of these parameters at different stages of pathology. The main finding of the study was that the brain concentration of [^124^I]8D3-F(ab’)_2_-h158 increased with degree of Aβ pathology. Once Aβ started to accumulate in the brains of tg-ArcSwe and tg-Swe mice, there was no overlap in brain concentrations of [^124^I]8D3-F(ab’)_2_-h158 between transgenes and wt mice (Figs 3 and 4), confirming [^124^I]8D3-F(ab’)_2_-h158 as a specific radioligand to follow progression of Aβ pathology. The histopathological analyses also confirmed the spatial distribution of pathology, i.e. abundant Aβ accumulation in striatal, cortical and hippocampal regions which also showed high levels of [^124^I]8D3-F(ab’)_2_-h158.

Age or genotype did not influence systemic pharmacokinetics or biodistribution to major organs. Thus, this finding confirms that [^124^I]8D3-F(ab’)_2_-h158 signal detected with PET in the brain of transgenic animals is specific to Aβ pathology. The two organs that showed the highest radioligand concentrations 72 h after administration, i.e. directly after completion of the PET scan, were spleen and bone. TfR is expressed on reticulocytes (immature red blood cells), which are found in high concentrations in bone marrow and spleen. Although much higher levels were found in these two organs compared to the other studied peripheral organs, the [^124^I]8D3-F(ab’)_2_-h158 concentration in brain in old transgenes was similar to that found in bone. Binding to reticulocytes has previously been reported as a potential safety problem^22^, but with the low doses used for imaging, this is unlikely to be a concern for PET. Altered expression of TfR in the brain caused by Aβ accumulation could in theory confound the interpretation that increased [^124^I]8D3-F(ab’)_2_-h158 levels are due directly to increased Aβ in the brain. However, we have previously shown that 8D3-F(ab’)_2_-h158 can enter the brain equally well in transgenic and wt animals, but is only retained in the brains of transgenic mice. Further, to rule out the possibility that [^124^I]8D3-F(ab’)_2_-h158 visualizes TfR rather than Aβ, we also generated a bispecific fusion protein based on 8D3 and a F(ab’)_2_ fragment of an antibody lacking an intra-brain target and showed that this antibody has the ability to cross the BBB but is not retained in the brains of either transgenic or wt mice 3 days post injection^17^.

PET data is often quantified as the radioactivity ratio of a region of interest to a reference region^23^. The reference region should be devoid of any specific binding. The abundant use of reference region methods in PET is first of all a means to avoid blood sampling but the methods are also good for normalizing the data for differences in scanner calibrations, differences in systemic pharmacokinetics or if the amount of radioactivity administered to the subject under investigation is unknown. In addition, the reference region, with no specific binding, serves as an estimate of the concentration of the radioligand in all brain regions that is available for interaction with specific binding sites. The reference region should be unaffected by the disease state. In AD (and in many studies of receptor function) the cerebellum or parts of the cerebellum is chosen as a reference region. We have previously shown that cerebellum seems to be a good choice of reference region up to a certain stage of pathology^17^ and the present study supports this conclusion. However, while the PET and *ex vivo* measures of the brain-to-cerebellum ratio of [^124^I]8D3-F(ab’)_2_-h158 increased up to 18 months, we now found that it then remained stable or decreased in older animals, especially in the tg-ArcSwe model. The reason for the observed stabilization of the ratio seemed to be twofold. First, in the tg-ArcSwe model it was a consequence of emerging Aβ pathology also in the cerebellum (Figs 4 f and 5). Second, both PET images and *ex vivo* measurements of [^124^I]8D3-F(ab’)_2_-h158 indicated that there was only limited pathology progression in the tg-ArcSwe brain between 18 and 24 month of age. The ratio in the tg-Swe mice also seemed to stabilize, although less evidently, despite the fact that both PET images and histopathological analysis indicated that Aβ pathology continued to progress in the brain between 18 months and 24 months. It is likely that soluble aggregates of Aβ, i.e. the target of [^124^I]8D3-F(ab’)_2_-h158, appear in the cerebellum even earlier than plaques and this may explain the stabilization of the ratio in tg-Swe mice. In summary, quantification using cerebellum as a reference may not be ideal at very advanced stages of disease progression. At this stage it may be more appropriate to normalize brain concentrations to blood concentrations of [^124^I]8D3-F(ab’)_2_-h158 (or other Aβ visualizing radioligands).

Several PET studies using currently available Aβ PET-ligands such as [^11^C]PIB, visualizing dense core plaques, have been conducted in animal models of Aβ pathology (for a review, see Brendel, *et al*.^24^). These studies have reported cortex-to-cerebellum concentration ratios of maximum 1.3-1.4, while information on cerebellum pathology has not been included^24,25^. In addition, animals harboring only the Swedish mutation leading to loosely structured plaques have displayed ratios of around 1, i.e. the same as seen in young transgenes and wt mice^24–26^. This can be compared to ratios of around 2, including mice with only the Swedish mutation, obtained with [^124^I]8D3-F(ab’)_2_-h158 at ages before pathology in cerebellum may interfere with the readout. The improved ‘dynamic range’ compared to previous Aβ PET-ligands and the selective binding to soluble aggregates of Aβ^17^, which are formed prior to Aβ plaques and are likely to be a more suitable therapeutic target, positions [^124^I]8D3-F(ab’)_2_-h158 as a promising tool for studying new therapeutic entities in the preclinical setting.

The present study, in line with previous preclinical studies of Aβ pathology, also points to the notion that the choice of animal model and the type of pathology in the chosen model will influence the readout and should therefore be carefully considered. The tg-Swe model showed higher PET ratios and higher concentrations of [^124^I]8D3-F(ab’)_2_-h158 in brain than tg-ArcSwe mice during advanced stages. This is in line with previous studies showing that tg-Swe mice, with a 6-fold over-expression of the human AβPP gene^27^, have more toxic soluble Aβ aggregates than tg-ArcSwe mice at an old age^17^ despite the fact that the later model showed more Aβ plaque pathology in the histological analyses (Fig. 5). We previously showed that the tg-Swe model displayed lower [^11^C]PIB binding than tg-ArcSwe mice, while the opposite was found for [^124^I]8D3-F(ab’)_2_-h158^17^. It therefore appears that antibody-based imaging with [^124^I]8D3-F(ab’)_2_-h158 quantifies soluble aggregated forms of Aβ while the [^11^C]PIB signal corresponds to dense core plaque pathology. Therapeutic efforts are presently largely aimed at decreasing the formation of new Aβ (inhibition of β- and γ- secretases) or removing toxic Aβ aggregates. Thus, histological stainings or even *in vivo* PET studies using [^11^C]PIB and analogues may not be the optimal tools to evaluate effects of these therapeutic strategies. Instead antibody-based imaging, especially antibody-based ligands targeting oligomers and protofibrils may be a more suitable option.

In a longer and more general perspective the current study showed that antibody-based radioligands can be utilized for imaging of targets within the central nervous system. Development of small molecular PET ligands, especially for misfolded proteins found in neurodegenerative diseases such as AD and Parkinson’s disease, has been hampered by substantial off-target binding. Antibodies can be highly specific for their target protein and may therefore be an attractive alternative to small molecular ligands as long as they can be engineered to reach the brain parenchyma.

In conclusion, this study demonstrates that antibody based PET imaging is a sensitive and dynamic method for *in vivo* assessment of Aβ pathology in AD transgenic mice and may become a valuable tool for disease staging of AD patients and to monitor effects of Aβ directed treatment.

## Methods

### Generation of a bispecific 8D3-F(ab’)_2_-h158

The bispecific fusion protein 8D3-F(ab’)_2_-h158 was generated as described previously^17^. In short, BAN2401^28^, a humanized form of mAb158 that presently is studied in clinical trials as anti-Aβ treatment, was cleaved with bacterial enzyme IdeS^29^, manufactured and distributed as FabRICATOR (Genovis AB, Lund, Sweden), to generate a F(ab’)_2_ fragment. F(ab’)_2_-h158 and transferrin receptor antibody 8D3^30^ (AbD Serotec, Oxford, UK) were then chemically conjugated with the Solulink technology (Solulink protein conjugation kit; Solulink, San Diego, CA, USA), where each of the conjugated proteins is modified with one of two linkers, which bind specifically to each other to make a permanent bond. To purify the fusion protein, the preparation was incubated for 1 h with Capture Select anti-Fc (multi-species) resin (Invitrogen) to specifically deplete the preparation of unconjugated F(ab’)_2_ fragments, which lack the Fc domain. After elution from the resin, the preparation was incubated for 1 h with Capture Select anti-CH1 resin (Invitrogen), which specifically binds to the constant domain 1 of human IgG heavy chain, thus depleting the sample of unconjugated 8D3.

### Animals

Two transgenic mouse models maintained on a C57BL/6 background were used: the tg-Swe (n = 22) model harbouring the *Swedish* double mutation *(AβPP KM670/671NL)* and the tg-ArcSwe (n = 18) model with the Swedish and the *Arctic (AβPP E693G)* mutations. Tg-Swe mice have a fairly late onset of plaque pathology starting at 10–12 months of age and then increasing rapidly with age^31^. Tg-ArcSwe mice show elevated levels of soluble Aβ protofibrils already at a very young age and a developing plaque pathology starting at around 6 months of age^19,31,32^. Tg-Swe mice display fairly loose and unstructured plaques while tg-ArcSwe mice exhibit dense core Aβ plaque pathology resembling plaques often found in the human AD brain. Both males and females were used and littermates were used as wild-type control animals (wt, n = 18). The animals were housed with free access to food and water in rooms with controlled temperature and humidity in an animal facility at Uppsala University. All experimental procedures and protocols were performed in accordance with the relevant guidelines and regulations and the study protocol was approved by the Uppsala Animal Experimental Ethical Committee (#C216/11, #C110/11 and #C17/14).

### Radiochemistry and analysis of the radioligand

The bispecific fusion protein 8D3-F(ab’)_2_-h158 was radiolabeled with iodine-124 (^124^I) using direct radioiodination^17,33^. Briefly, 58 µl ^124^I stock solution (Perkin-Elmer Inc.) was pre-incubated 10 min with 12 µl 50 µM NaI before addition of 260 pmoles of fusion proteins and 40 µg Chloramine-T, mixed in PBS to a final volume of 420 µl. The reaction was quenched by addition of 80 µg of sodium metabisulfite after 120 s. Free iodine and low-molecular weight components were separated from 8D3-F(ab’)_2_-h158 with a disposable NAP-5 size exclusion column (GE Healthcare AB, Uppsala, Sweden) according to the manufacturer’s instructions (cut-off 5 kDa). 8D3-F(ab’)_2_-h158 was then eluted in 1 ml of PBS. The yield was calculated based on the added radioactivity and the radioactivity in the purified radioligand solution. Radiochemical purity and stability of the ligand after three days’ incubation in mouse blood plasma was assessed with instant thin-layer chromatography (ITLC). Approximately 1 µl of the radiolabeled ligand was applied to a chromatography strip (Biodex, Shirley, NY, USA) and put into 70% acetone followed by exposure to positron-sensitive phosphor screens (MS, MultiSensitive, PerkinElmer, Downers grove, IL, USA). Images were acquired with a Cyclone Plus Imager system (Perkin Elmer) and data were analyzed with OptiQuant image analysis software (Packard Instrument Co., Meriden, CT, USA). Labelling was always performed less than 2 h prior to each study. Affinity for Aβ protofibrils and/or TfR was tested with ELISA^17^ on the same day as the labelling and the start of the study.

### PET

The day before injection of [^124^I]8D3-F(ab’)_2_-h158, animals were given water supplemented with 0.2% NaI to reduce thyroidal uptake of ^124^I. PET and CT scans were acquired at 72 h post injection of [^124^I]8D3-F(ab’)_2_-h158. Prior to the start of the PET scan, the animal was anaesthetised with isoflurane (2.5% in medical air) and placed on the pre-heated scanner bed of the animal PET/CT scanner (Triumph Trimodality System, TriFoil Imaging, Inc., Northridge, CA, USA). Anaesthesia was maintained during the whole scan with isoflurane (1.5–2.0%). PET data was collected in list mode for 60 min followed by a CT examination for 3 min (Field of View (FOV) = 8.0 cm).

The PET data was reconstructed using a MLEM 2D algorithm (10 iterations). The CT raw files were reconstructed using Filter Back Projection (FBP). All subsequent processing of the PET and CT images were performed in imaging software Amide 1.0.4^34^. The CT scan was manually aligned with a T2 weighted, MRI based mouse brain atlas^35^ containing outlined regions of interests for hippocampus, striatum, thalamus, cortex and cerebellum. The CT, including the outlined regions of interest, was then aligned with the PET, thus transferring the regions of interest to the PET image. The PET data was quantified as a concentration ratio of the radioactivity in 6 regions of interest (cortex, hippocampus, thalamus, striatum, whole brain and midbrain which is a summation of the first 4 regions) to that in cerebellum.

### *Ex vivo* analyses

Blood samples (8 µl) were obtained from the tail vein at 0.5, 1, 2, 3, 4, 6, 8, 24 and 48 h following administration of [^124^I]8D3-F(ab’)_2_-h158. A terminal blood sample was obtained from the heart after PET scanning at 72 h post injection. Brain, heart, liver, lung, kidney, spleen, pancreas, femoral bone and muscle were isolated after intracardiac perfusion with 50 ml physiological saline during 2 min. The left hemisphere of the brain was divided into two parts; cerebellum and the rest of the brain.

Radioactivity in blood samples and isolated organs was measured with a well counter (GE Healthcare, Uppsala, Sweden). The brain, cerebellum and blood concentrations, quantified as % of injected dose per gram tissue (% ID/g), were calculated as following:

1 $$ \% ID/g=Measured\,radioactivity\,per\,gram\,tissue\,(or\,blood)/Injected\,radioactivity$$

By dividing *%ID/g* in brain with the *%ID/g* in cerebellum a ratio similar to the tissue ratio obtained when quantifying PET data could be obtained. In addition, brain (or cerebellum) to blood ratios were also obtained for all animals by dividing the tissue concentration with the concentration in blood.

The half-life in blood was obtained by fitting an exponential curve to the data and then using the estimated elimination constant (k_el_) to calculate the half-life (t_1/2_ = ln(2)/k_el_).

### Histopathological analyses

Amyloid-beta pathology was studied on sagittal cryosections (20 µm) of the right hemisphere immunostained for Aβ40 (Agrisera, Umeå, Sweden) and with Amytracker^TM^ 545 (Ebba Biotech, Solna, Sweden).

For Aβ40 immunohistochemistry, sections were fixed in 4% PFA in PBS for 20 min at room temperature, washed in PBS and then incubated 40 min in pre-heated citrate buffer (25 mM, pH 7.3, 100 °C at the start of the procedure) for antigen retrieval. Sections were then transferred to 70% formic acid for 5 min at room temperature and washed under a constant flow of fresh MQ-H_2_O for another 5 min. Endogenous peroxidase activity was blocked with DAKO peroxidase block (Agilent Technologies, Kista, Sweden) during 15 min and permeabilized in 0.4% triton in PBS for 5 min. After 10 min in DAKO-block (0.25% casein in PBS) to inhibit unspecific binding, sections were incubated overnight with 0.5 µg/ml polyclonal anti-Aβ40 antibody (Agrisera, Umeå, Sweden), followed by a 30 min incubation with 5 µg/ml biotinylated goat anti-rat (Vector Laboratories Inc., Burlingame, CA) and 30 min with Streptavidin-HRP (Mabtech AB). The staining was developed with NOVA RED chromogen (Vector Laboratories Inc.) for 10 min on a shaker and then washed in MQ-H_2_O for 1 min and quickly dipped first in 95% EtOH and then 99.9% EtOH. Sections were air-dried, mounted with DPX mounting medium (Sigma Aldrich, Sweden) and analyzed with Nikon microscope (DXM1200F, Nikon Instruments Inc., Melville NY, USA).

For Amytracker staining of Aβ aggregates, sections were fixed with cold (−20 C) ethanol and rehydrated in a mix of deionized water and ethanol (1:1) for 5 min at room temperature. After 5 min PBS incubation, Amytracker^TM^ 545 was applied (1:1000) and incubated for 30 min at room temperature. Tissue sections were then washed 3 × 5 min in PBS and mounted with Vectashield Hard Set with DAPI (Sigma Aldrich, Sweden). Pictures were captured with a Zeiss Axiolmager Z1 microscope (LMS700) using FITC filter set. The Zen 2012 software was used for further image processing.

### Statistics

Results reported are presented as mean ± standard deviation. Data was analyzed with two-way ANOVA followed by Bonferroni’s *post hoc* test. Pearson’s correlation was used to analyze correlation of brain concentration of [^124^I]8D3-F(ab’)_2_-h158 and age or Aβ protofibril concentration. All analyses were performed in GraphPad Prism 6.0 (GraphPad Software, Inc, La Jolla, CA, USA).

### Data availability

The datasets generated during the current study are available from the corresponding author on reasonable request.
